# Advice to the FDA to Improve Its Proposed Guidelines to Rationalize Clinical Trials by Restricting Placebo Control, Preventing Low-Powered Studies, and Disallowing Studies Where Bioavailability Is Not Proven

**DOI:** 10.3390/ph17111424

**Published:** 2024-10-24

**Authors:** Sarfaraz K. Niazi

**Affiliations:** College of Pharmacy, University of Illinois, Chicago, IL 60612, USA; sniazi3@uic.edu; Tel.: +1-312-297-0000

**Keywords:** clinical trials, FDA, multi-regional trials, integrated RCT in clinical practice, adaptive trials, decentralized trials, real-world trials, remote trials, placebo, study power, bioavailability, biosimilars

## Abstract

Randomized controlled trials (RCTs) are the gold standard for testing the safety and efficacy of new drugs and biologicals. The US Food and Drug Administration (FDA) has proactively improved the trial designs to make them scientifically rational while avoiding unnecessary human exposure. Several new guidelines by the FDA have come in 2024 that address consolidating the RCTs with the Real-World Evidence (RWE) trials, decentralizing the testing platforms, and allowing the point-of-use clinicians to participate. However, the issue of placebo control remains, which is part of RCTs, and it should be reduced or removed given the organic impact of placebo that compounds the efficacy evaluation (explanatory trials), as opposed to effectiveness trials (pragmatic trials), which measure the degree of beneficial effects in “real-world” clinical settings. Additionally, clinical trials with low study power should be allowed, and when the proof of bioavailability at the site of action is not present, it should be removed. It is advised that the FDA issue a comprehensive guideline to consolidate its several guidelines and consider the role of placebo in making drug development a more affordable exercise while meeting the requirement to minimize the abuse of humans in such trials.

## 1. Introduction

The history of clinical trials can be traced back to the early 18th century [1]. James Lind conducted one of the first documented clinical trials in 1747, when he tested the effects of different dietary treatments on sailors suffering from scurvy. Lind’s trial involved 12 sailors, divided into groups, and provided with other supplements, including citrus fruits, which led to the discovery that vitamin C could prevent scurvy [2].

In the 19th century, the scientific method became more prominent in medicine, leading to more structured experiments. By the mid-1800s, the placebo concept was introduced by physician John Haygarth, who used “sham treatments” to assess their impact compared to actual interventions [3].

The modern era of clinical trials began in the early 20th century with the introduction of the randomized controlled trial [4]. In 1948, the British Medical Research Council conducted one of the first RCTs to evaluate the effectiveness of streptomycin for tuberculosis treatment. This trial established key principles of randomization and control, which are still fundamental to clinical trials today [5].

The double-blind design, where neither participants nor researchers know who receives the treatment or placebo, emerged in the 1950s, reducing biases and increasing the reliability of trial results. This approach was pivotal in developing safe and effective treatments during the 20th century [6].

The 1960s saw a significant shift in the regulation of clinical trials following the thalidomide disaster. Thalidomide, a drug given to pregnant women for morning sickness, caused severe congenital disabilities. This tragedy highlighted the need for stricter drug testing and led to the creation of the Kefauver–Harris Amendment to the U.S. Food, Drug, and Cosmetic Act in 1962. This law required drugs to be proven safe and effective through clinical trials before being marketed [7].

By the 1980s and 1990s, global collaboration on clinical trial standards intensified. The formation of the International Council for Harmonisation of Technical Requirements for Pharmaceuticals for Human Use (ICH) in 1990 established global guidelines for clinical trials, focusing on safety, efficacy, and quality. The ICH E6 Good Clinical Practice (GCP) guidelines, published in 1996, remain a cornerstone of regulatory standards today, ensuring that clinical trials are scientifically sound and ethically conducted [8].

As technology advanced, so did clinical trials. Adopting electronic health records, telemedicine, and wearable health devices has transformed how data are collected and monitored during trials. These innovations have allowed for more decentralized trials, where participants can engage in clinical research from remote locations, reducing logistical barriers [9].

Regulators and researchers have recently pushed for more flexibility in clinical trial designs. The COVID-19 pandemic accelerated these changes, as the need for rapid vaccine development demanded new approaches. Regulators like the FDA and EMA issued guidance allowing remote monitoring, telemedicine, and adaptive trial designs, where trials can be modified based on interim data without compromising scientific rigor [10].

## 2. Trial Types

Clinical trials have evolved significantly from the early days of James Lind’s scurvy experiments to today’s complex, technology-driven trials. Regulatory frameworks, from the Kefauver–Harris Amendment to the ICH guidelines, have provided crucial oversight, ensuring the safety and efficacy of new treatments. As the world moves towards more decentralized and adaptive trial designs, clinical research continues to progress, driven by technological advances and the lessons learned from global health crises like the COVID-19 pandemic.

### 2.1. Remote Trials

Allowing more trials to be conducted remotely can increase patient diversity and participation [11]. In September 2024, the FDA issued a final guidance, “Conducting Clinical Trials with Decentralized Elements”, providing recommendations for sponsors, investigators, and other interested parties regarding implementing decentralized elements in clinical trials [12].

### 2.2. Adaptive Trial Designs

Permitting more flexibility in trial protocols to modify endpoints, doses, or patient groups based on early findings, improving efficiency [13]. Adaptive clinical trials are a type of clinical study design that allows for modifications to trial procedures based on accumulating data during the study without compromising the validity and integrity of the results. Adaptive trials are flexible, unlike traditional clinical trials, which have a fixed design from start to finish. They can adjust aspects such as dosing, patient allocation, or sample size while the trial is ongoing. This flexibility can lead to more efficient use of resources, faster decision making, and potentially reduce the time and cost of drug development [13].

One of the primary features of adaptive trials is the pre-specified adaptation rules, which are defined before the trial begins. These rules ensure that any changes made are driven by data, minimizing bias and preserving the trial’s statistical rigor. For instance, interim analyses may determine if certain patient groups respond better to the treatment, allowing researchers to allocate more patients to that group or stop enrolling patients into less effective groups [14].

Adaptive trials have been used in various therapeutic areas. One well-known example is the I-SPY 2 trial, which evaluated multiple treatments for breast cancer. The trial used a platform design to simultaneously test different therapies, adapting based on which treatments showed the most promise in patients with specific biomarkers. This allowed researchers to quickly identify effective therapies while dropping ineffective ones [15]. Adaptive trials were also pivotal during the COVID-19 pandemic, such as in the RECOVERY trial in the UK, which evaluated multiple treatments for COVID-19. The trial was able to adapt its protocols to add or remove treatments based on interim results [16].

The application of adaptive clinical trials extends to improving the efficiency of drug development, particularly in complex conditions with high variability in patient response. They are increasingly utilized in personalized medicine, where patient subpopulations may respond differently to treatments. This trial design can provide faster access to promising therapies and more accurate data on treatment efficacy, ultimately benefiting both patients and pharmaceutical companies [14,17].

Incorporating data from real-world healthcare settings (e.g., electronic health records) helps supplement traditional clinical trials [18].

### 2.3. Real-World Evidence (RWE)

The RWE Program was established to evaluate how RWD can be reliably used to generate evidence of product effectiveness, guide regulatory submissions, and expand labeled indications for approved therapies. Since its inception, the program has focused on advancing RWE through pilot projects, stakeholder engagement, and the development of methodological standards to ensure data quality and integrity. The FDA’s guidance documents outline best practices for designing and conducting RWE studies, emphasizing transparency and rigorous data validation processes to support regulatory submissions.

Traditional clinical trials, often randomized controlled trials (RCTs), are designed with strict protocols, controlled environments, and highly selected patient populations to minimize bias and establish causal relationships between an intervention and outcomes. These trials typically involve randomly assigning patients to treatment or control groups, and outcomes are measured under tightly regulated conditions, often not reflecting real-world settings.

The 21st Century Cures Act (Cures Act) aims to accelerate medical product development and bring innovations faster and more efficiently to the people who need them most by capitalizing, among other things, on using RWE. In response to the Cures Act, which added section 505F to the Federal Food, Drug, and Cosmetic Act (FD&C Act) (21 U.S.C. 355g), relating to the use of RWE in regulatory decision making, the FDA created an RWE Program to evaluate the use of RWE to support the approval of new indications for drugs already approved under section 505(c) of the FD&C Act (21 U.S.C. 355(c)) or to help to support or satisfy post-approval study requirements. The RWE Program also covers biological products licensed under section 351(a) of the Public Health Service Act.

This approach can reduce recruitment costs, as participants are drawn from existing patient populations, bypassing the often expensive and lengthy process of identifying eligible trial participants. Recruitment costs typically account for 30% of a clinical trial’s total expenses, meaning using RWE can lead to savings of USD 20–30 million for large trials, which can cost around USD 100 million [19].

Additionally, RWE-based trials often take less time because researchers can leverage existing data rather than waiting for participants to progress through various trial stages. Traditional trials can take 5–7 years from Phase I to FDA approval but using RWE can potentially reduce this timeline by 1–2 years, especially for post-approval studies. For example, Phase IV studies that typically take years can be completed in a few months using RWE, allowing for quicker regulatory decisions. The U.S. Food and Drug Administration (FDA) encourages using RWE for label expansions, reducing timelines for certain drug approvals [18].

This could cut data collection costs by as much as 50%, translating to savings of USD 10–20 million for large trials. Furthermore, RWE enables more efficient trial designs by allowing for smaller, more targeted sample sizes, as real-world data often captures a more diverse and representative patient population. Reducing sample sizes by 10–20% could save millions in recruitment and operational costs [20].

RWE also facilitates faster regulatory approvals. Using RWE to gain approval for additional drugs already approved for one indication can save 1–2 years in the regulatory process. This is particularly important for expanding drug labels or post-approval studies where real-world data can supplement clinical trial data. One notable example is Pfizer’s use of RWE for a label expansion for Ibrance (palbociclib) for male breast cancer. Pfizer relied on insurance claims and EHRs to secure FDA approval in a fraction of the time it would have taken for a complete Phase III randomized trial, saving an estimated 2–3 years and millions in trial costs [21].

While increasingly accepting RWE, regulatory bodies still require rigorous data validation, which can involve additional costs for auditing and quality control [22]. In summary, while RWE has the potential to reduce clinical trial costs by 10–50% and shorten trial timelines by 1–3 years, these benefits depend on the quality of the data and the regulatory framework in place. RWE is becoming an increasingly valuable tool in drug development and is helping to modernize clinical trial processes in ways that reduce both cost and time while maintaining high safety and efficacy standards.

### 2.4. Biosimilars Trials

Several biosimilars have been tested using Real-World Evidence (RWE) to supplement clinical trial data and provide additional insights into their safety, efficacy, and cost-effectiveness in broader patient populations. RWE has been particularly valuable in demonstrating that biosimilars perform similarly to their reference biologics in real-world settings outside of controlled clinical trials. Below are a few examples of biosimilars that have been evaluated using RWE, include Infliximab (Remsima/Inflectra) for Inflammatory Bowel Disease (IBD) [23], Rituximab (Truxima/Rixathon) for Rheumatoid Arthritis and Lymphoma [24], Trastuzumab (Herzuma/Kanjinti) for Breast Cancer [25], Epoetin Alfa (Epoetin Biosimilars) for Anemia in Chronic Kidney Disease [26], and Filgrastim (Zarxio/Nivestim) for Neutropenia in Chemotherapy Patients [27]. Prominent RWE studies include:

- Supporting Expanded Use of Ibrutinib in Chronic Lymphocytic Leukemia (CLL) [28]
- Diabetes Management—Dapagliflozin and Cardiovascular Outcomes [29]
- Oncology—Pembrolizumab for Advanced Non-Small Cell Lung Cancer [30]
- Cardiovascular Outcomes in Anticoagulant Use [31]
- Multiple Sclerosis Treatment—Ocrelizumab [32]
- COVID-19 Treatments—Tocilizumab [33]
- Alzheimer’s Disease—Aducanumab [34]

Post-market evaluations are frequently used to adjust the dosages of drugs. It should be a reasonable assumption that with such vast data on the safety and efficacy of biosimilars, the regulatory agencies should remove the clinical testing requirements [35].

### 2.5. Randomized Clinical Trials

Clinicians and policymakers often distinguish between an intervention’s efficacy and effectiveness. Efficacy trials (explanatory trials) determine whether an intervention produces the expected result under ideal circumstances. Effectiveness trials (pragmatic trials) measure the degree of beneficial effects in “real-world” clinical settings and yield the most relevant evaluation of a therapy: how it works in the real world.

Integrating Randomized Controlled Trials (RCTs) into routine clinical practice can significantly alter traditional RCTs’ cost, time, and structure. Traditionally, RCTs are conducted in controlled environments, requiring specialized infrastructure, dedicated staff, and extensive patient monitoring. These factors contribute to traditional trials’ high costs and extended timelines. By embedding RCTs into routine clinical care, much of the infrastructure needed for site management and trial operations can be shared with existing healthcare settings, such as hospitals and clinics where patients are already receiving treatment. This reduces logistical costs, particularly those associated with site management, by an estimated 20–30% in large multi-site trials [36]. Moreover, because routine clinical care already involves using electronic health records (EHRs) and other real-time data collection methods, integrating trials into these systems can further reduce data management and monitoring costs, potentially cutting data collection expenses by up to 25% [22].

One of the significant benefits of integrating RCTs into routine practice is the potential for faster recruitment. Traditional trials often face lengthy recruitment periods due to strict inclusion/exclusion criteria and the need for specialized screening processes. In contrast, integrating RCTs into routine care allows researchers to recruit patients directly from those already receiving treatment, thus significantly reducing recruitment timelines. In some cases, recruitment times can be reduced from months to weeks, which may shorten overall trial timelines by 1–2 years [19]. Additionally, recruiting patients from a real-world setting increases the diversity of trial participants, allowing for a more representative sample, including patients with comorbidities or those outside the typical parameters of clinical trials. This increased generalizability can improve the external validity of the trial outcomes [37].

Collecting real-time data as part of routine healthcare is another area where integrating RCTs into clinical practice can lead to savings. In traditional RCTs, patients must frequently visit trial sites for follow-ups and monitoring, requiring substantial investment in time and resources. By contrast, in trials integrated into routine practice, patient data can be collected during standard care visits, leveraging pre-existing healthcare records and EHRs [20]. This approach eliminates the need for additional visits solely for data collection and allows researchers to gather comprehensive data without the overhead of dedicated trial appointments. This real-time data collection and the ability to access larger datasets through EHRs streamline the process and reduce overall trial costs.

While integrating RCTs into routine practice shares similarities with Real-World Evidence (RWE) methodologies, fundamental differences remain. Unlike observational studies that characterize RWE, integrated RCTs maintain the element of randomization, preserving the ability to establish causality. This randomization ensures that integrated RCTs retain the scientific rigor of traditional trials while benefiting from the flexibility and broader applicability of RWE. In traditional RWE studies, treatments are not randomized, and outcomes are observed in the context of standard care. While RWE is invaluable for supplementing trial data or exploring post-market safety and effectiveness, it is often less likely to be used for initial drug approvals [18].

Despite the efficiencies of integrating RCTs into routine clinical practice, it is essential to note that this approach does not eliminate the need for structured and organized trials. Regulatory agencies, such as the U.S. Food and Drug Administration (FDA) and the European Medicines Agency (EMA), still require well-defined protocols, randomization, and ethical oversight to ensure that trials meet the necessary scientific and regulatory standards. Integrating RCTs into routine practice represents a hybrid model, blending the rigor of traditional trial design with the efficiencies of real-world data collection. It allows for more flexible, decentralized trials, but critical elements of trial structure—such as randomization, control, and data integrity—remain essential to the study’s success [20].

In conclusion, integrating RCTs into routine clinical practice offers substantial potential to reduce both the cost and time of clinical trials by streamlining recruitment, data collection, and site management. This approach retains the rigor of randomization and control, distinguishing it from purely observational RWE studies while leveraging the efficiencies of real-world healthcare settings. As the regulatory landscape evolves, this hybrid model will likely become increasingly valuable in drug development, especially as the FDA and EMA support decentralized trial methodologies [38].

## 3. FDA New Guidelines

The US FDA has been at the forefront of rationalizing clinical trials, driven by the legislation to prevent human abuse in testing drugs [39,40]. Most recently, the FDA has issued two guidelines that allow a more rational approach to testing new drugs. Still, it will also significantly affect the cost of developing new drugs.

### 3.1. Decentralized Trials

Decentralized elements allow trial-related activities to occur remotely at convenient locations for trial participants. Decentralized elements may include telehealth visits, in-home visits with remote trial personnel, or local healthcare providers. In this guidance, a decentralized clinical trial (DCT) refers to a clinical trial that includes decentralized elements where trial-related activities occur at locations other than traditional clinical trial sites. By enabling remote participation, DCTs may enhance convenience for trial participants, reduce the burden on caregivers, expand access to more patient populations, improve trial efficiencies, and facilitate research on rare diseases and diseases affecting populations with limited mobility. This guidance is a part of the FDA’s commitment to advance innovation in clinical trial design and conduct.

The document titled “*Conducting Clinical Trials With Decentralized Elements: Guidance for Industry, Investigators, and Other Interested Parties*” [12], issued in September 2024 by the FDA, offers comprehensive guidelines for incorporating decentralized elements into clinical trials. It defines decentralized clinical trials (DCTs) as trials where activities occur remotely, away from traditional sites, to enhance participant convenience and broaden trial access. The guidance emphasizes the FDA’s regulatory requirements for decentralized trials, covering telehealth visits, local healthcare providers (HCPs), and digital health technologies (DHTs).

This guideline outlines the role of sponsors and investigators in ensuring trial design limits the variability in data collection, including specifying which visits are conducted in person versus which were remote. It discusses potential challenges in remote assessments, such as introducing bias and variability, and emphasizes the importance of patient input in trial design to address these issues. Digital health technologies are highlighted to collect data remotely and improve access, especially for underserved populations.

Roles and responsibilities are clarified, particularly regarding the sponsor’s duty to coordinate multiple data sources and local HCPs and ensure compliance with local laws. Investigators are held accountable for protecting participants’ safety, supervising trial-related activities, and ensuring accurate data reporting. The guidance covers key areas like FDA oversight, informed consent processes, investigational product management (including drug and device considerations), packaging, and shipping requirements.

The document stresses the importance of safety monitoring and risk management, advising that safety plans be adapted to decentralized environments, with provisions for reporting adverse events and handling investigational products in remote settings. It underscores that all electronic systems used in DCTs must comply with FDA’s data reliability, security, and privacy regulations.

Lastly, the guidance provides a glossary of key terms, including definitions for decentralized clinical trials, digital health technologies, and investigational products. It advises that sponsors and investigators ensure the proper coordination of decentralized elements to improve access, enhance data quality, and safeguard trial participants.

In the context of clinical trials, “traditional sites” refer to established physical locations where trial-related activities are typically conducted. These include hospitals, clinics, research institutions, or other designated medical facilities where participants visit for in-person interactions with trial personnel, receive investigational treatments, undergo monitoring, and participate in assessments.

At traditional sites, clinical trial staff, such as investigators, nurses, or other healthcare professionals, are present to supervise and administer various aspects of the trial. These sites are designed to facilitate controlled and standardized data collection, ensure participant safety through direct supervision, and adhere to regulatory protocols under centralized oversight.

In contrast, decentralized clinical trials (DCTs) allow some or all these activities to occur outside of these traditional settings, such as in participants’ homes, via telehealth, or at local healthcare providers’ facilities, broadening accessibility and reducing the burden on participants.

### 3.2. Multiregional Clinical Trials (MRCTs)

In September 2024, the FDA issued a new draft guideline on Conducting Multiregional Clinical Trials in Oncology [41]. The new draft guidance, “*Considerations for Generating Clinical Evidence from Oncology Multiregional Clinical Development Programs*”, expands on current principles for MRCTs and, when finalized, will provide additional recommendations to improve the planning, design, conduct, and analysis of future oncology MRCTs. It will also aid sponsors in planning multiregional clinical development programs that consider the agency’s evaluation of trial results that can be applied to the intended use population in the U.S. and U.S. standard oncological care.

Multiregional clinical trials (MRCTs) and decentralized clinical trials (DCTs) are distinct approaches, but they can be integrated depending on the trial design. MRCTs are conducted across multiple geographic regions or countries to gather safety and efficacy data from diverse populations, typically using traditional centralized trial sites. In contrast, DCTs leverage technology and remote methods, such as virtual visits, mobile health devices, and telemedicine, to reduce the need for participants to visit central locations. These decentralized elements can enhance MRCTs by improving patient recruitment, retention, and logistical efficiency, especially in remote or underrepresented areas. By incorporating decentralized tools like remote monitoring and telemedicine into an MRCT, the trial can overcome geographic barriers and make participation more convenient while maintaining the diverse population data that MRCTs aim to collect. While MRCTs and DCTs have different focuses—MRCTs on regulatory diversity and DCTs on participation flexibility—the two can be effectively combined to create a hybrid model that maximizes both strengths.

## 4. Integrating Randomized Controlled Trials for Drug and Biological Products into Routine Clinical Practice

As part of the FDA’s Real-World Evidence (RWE) Program, this guidance is intended to support the conduct of randomized controlled drug (meaning chemical and biological entities) trials (RCTs) with streamlined protocols and procedures that focus on essential data collection, allowing integration of research into routine clinical practice. Such trials have sometimes been referred to as *point-of-care* or *large simple trials*. Like decentralized clinical trials [12], which aim to bring trial-related activities to patients’ homes or other convenient locations, such RCTs may improve convenience and accessibility for participants and allow for enrollment of more representative populations, resulting in more generalizable trial results. Leveraging established healthcare institutions and existing clinical expertise in the medical community can reduce startup times and speed up enrollment.

Traditional randomized controlled drug trials typically capture much patient information at baseline and throughout the trial. Healthcare professionals may also collect these data in routine clinical practice patient interactions. Researchers may be able to use those data to satisfy trial data requirements, reducing the need for dedicated trial sites and duplicative data entry. This may improve convenience and accessibility for participants and allow for enrollment of more representative populations, resulting in more generalizable trial results.

Depending on the condition and the intervention to be studied, the spectrum of trial designs may range from those that are almost entirely reliant on data acquired by the participant’s local healthcare providers (HCPs) during routine clinical practice visits (either in person or virtually) to those that require significant supplementation with dedicated, research-specific activities for data collection conducted by trial staff. The contribution of local HCPs involved in clinical care and the contribution of dedicated trial staff may also vary, depending on the needs of the trial.

During routine clinical practice visits, local HCPs are often engaged by organizations to perform clinical activities that are not required as part of routine clinical care, such as insurance or employment medical examinations, medical examinations for driver’s licenses, or medical examinations for visa applications for travelers. Examples of activities local HCPs can conduct for these organizations include obtaining a medical history, conducting a physical examination, and performing a diagnostic procedure. These activities do not require practitioners to receive special training beyond their specialties or have a detailed knowledge of why the information is being requested. Suppose practitioners find an abnormality during these activities. In that case, they typically record the findings and ensure appropriate clinical management (e.g., referring the participant to their local HCP if they are not the patient’s HCP).

Similarly, sponsors may engage local HCPs (either directly or through clinical investigators or health care institutions) to perform certain clinical activities that are not required as part of routine clinical care but might be needed for a clinical trial, such as conducting a routine physical examination, ordering a chest radiograph, ordering a blood test at protocol-specified intervals, or collecting protocol-required information such as medical histories or outcomes. Sponsors should consider the complexity of trial requirements, the need for standardization of trial-related activities, and the need for research-specific expertise when deciding on the feasibility of trials in a practice setting. Integrating RCTs into clinical practice should not interfere with the appropriate delivery or administration of patient care.

As with traditional trials (i.e., those with only dedicated trial staff and sites), integrated RCTs into clinical practice may also seek to use real-world data from electronic or other health records to inform safety or effectiveness without the direct engagement of local HCPs. Such data include demographic information, prescription pharmacy data, diagnostic codes, and discharge summaries. The use of these data is covered in the guidance for industry issued in July 2024: Real-World Data: Assessing Electronic Health Records and Medical Claims Data to Support Regulatory Decision Making for Drug and Biological Products [42]. This guidance applies to studies involving FDA-approved drugs being studied for new indications, populations, routes of administration, or doses; drug safety studies for FDA-approved drugs; other post-marketing studies for FDA-approved drugs; comparative effectiveness studies for FDA-approved drugs; and trials of unapproved drugs when the safety profile is sufficiently characterized, and the drug is appropriate to be administered and managed in the setting of routine clinical practice. This guidance does not address non-interventional (observational) studies.

Traditional RCTs typically capture a large amount of protocol-specified patient information (e.g., patient characteristics, medical history, concomitant medications, vital signs, adverse events, laboratory results, measures of drug response, and clinical status) at baseline and throughout the trial. Some of these data are also collected in routine clinical practice. However, the specific procedures and methods, timing of collection, and documentation formats often differ from those in a clinical trial. Given the potential overlap in the information collected, data for clinical research can, under appropriate circumstances, be obtained from routine clinical practice interactions, reducing the need for dedicated trial sites.

There has been increasing interest in using real-world data acquired during routine clinical practice to support drug development. Advances in information technology and the widespread use of electronic health records (EHRs) have facilitated access to real-world data obtained during routine clinical care and provided new opportunities for integrating clinical research and clinical care. Institutions may be able to enhance the integration of clinical research and clinical care by designing EHR systems that capture health care information in standardized formats aligned with the format of information collected in case report forms used in RCTs.

Experience with trials conducted in clinical practice settings has demonstrated the potential value of this approach for drug development in certain circumstances. Such trials with simplified data collection have allowed rapid enrollment and evidence generation [43]. More recently, the widespread use of EHRs and other electronic data-gathering tools has made integrating clinical research and care more feasible. For example, in 2020, the RECOVERY trial was conducted using clinical practice infrastructure and local HCPs in hospitals throughout the United Kingdom. The trial results supported FDA approval of tocilizumab for treating hospitalized adult patients with COVID-19 who receive systemic corticosteroids and require supplemental oxygen, non-invasive or invasive mechanical ventilation, or extracorporeal membrane oxygenation [44].

This guidance describes the role of sponsors, clinical investigators, and local healthcare providers, using Quality by Design approach, identifying the trial population, obtaining informed consent, choosing suitable investigational drugs, randomization and blinding, comorbidities and concomitant medications, study endpoints, adverse events, especially where real-time monitoring of patients’ EHRs and periodic follow-up calls to study participants by trial personnel [45], data privacy, and inspections.

## 5. Cost

While various changes brought into clinical trials design are intended to reduce the unnecessary exposure to humans, the cost of trials remain uncertain and highly dependent on the drug type, the response variability, and the outcome analysis (Table 1).

## 6. Advice to the US FDA

The FDA has been proactive in bringing rationality to clinical trials, and it is anticipated that following these new guidelines, developers can significantly reduce the cost of these studies that contribute most of the development cost; however, there remain a few additional considerations that the FDA must address.

### 6.1. Placebo Control

**Figure 1:**
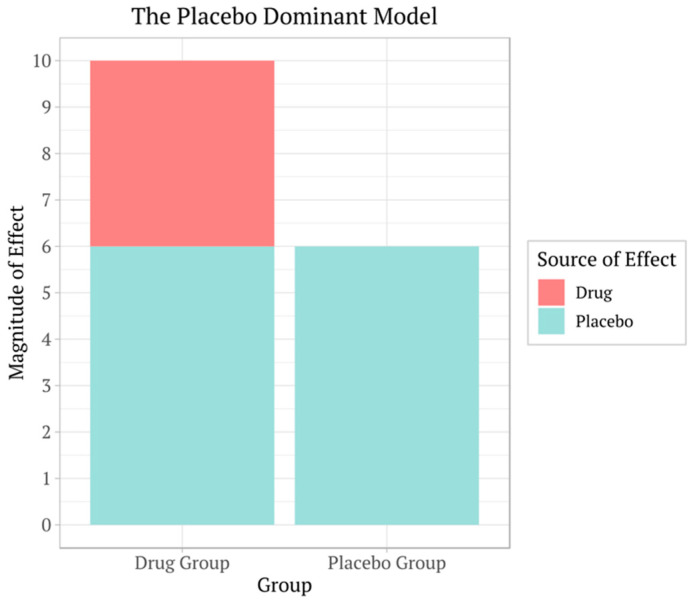
Clinical trials often compare the effects of a drug to a placebo to determine the drug’s effectiveness and check for side effects. In these trials, participants are randomly assigned to either receive the drug or an inactive placebo, and they usually do not know which they are getting. This is called a double-blind test, and it helps keep the research free from bias. By comparing the results from both groups, researchers can measure how the drug works and see if it is more effective than the placebo effect alone. Placebo effects can easily make the drug’s effects less prominent. Should a drug be approved for its net effect or compared to a placebo not in the patient’s use?

One main change required in clinical trial protocols is to decide whether a trial needs to be a controlled trial in the first place, referring to comparison with a placebo or a comparator product as this can significantly change the statistical power calculations (Figure 1).

A placebo intervention can bring about more than changes in perception; it can lead to organic changes mediated by the brain and genuine physiological changes impacting disease symptoms and progression. This dual mechanism underscores the complexity and power of the placebo effect in clinical practice and research. The placebo effect primarily involves the disease course in the context of diseases such as infections and cancer. In oncology, placebos mainly address symptoms and side effects rather than altering the disease course [46], because the concept of placebos affecting organic components is still unexplored. The brain’s release of endogenous opioids and other neurotransmitters in response to placebo treatments can modulate pain pathways, providing genuine pain relief that goes beyond mere perception. Additionally, placebo effects can activate brain regions associated with stress and immune responses, potentially influencing the body’s physiological state and improving symptoms [47].

Ethical analysis and international ethical guidance permit the use of placebo controls in randomized trials when scientifically indicated in four cases:

- When there is no proven effective treatment for the condition under study;
- Withholding treatment poses negligible risks to participants;
- When there are compelling methodological reasons for using a placebo, withholding treatment does not pose a risk of serious harm to participants;
- More controversially, when there are compelling methodological reasons for using a placebo, the research is intended to develop interventions that can be implemented in the population from which the trial participants are drawn. The trial does not require participants to forgo treatment they would otherwise receive.

Methodological reasons are essential for assessing the ethics of placebo controls in these controversial last two cases. Such studies are biased due to their smaller size, making them more subjective [48]. A systematic review and meta-analysis of 12,564 men found a significant association between placebo use and improved erectile function, with the effect size being larger among men with post-traumatic stress disorder [49]. Continuous outcomes, such as pain intensity measured on a scale, show more pronounced placebo effects than binary outcomes, such as the presence or absence of a symptom. This difference indicates that placebos might influence how patients perceive and report their symptoms rather than causing actual physiological changes [48]. Studies using three-armed trials [50] provide more precise insights into the placebo effect by isolating it through direct comparisons between placebo and no-treatment groups. These studies’ results suggest that placebos can noticeably impact patient-reported outcomes. Nevertheless, the effects are often modest and can diminish with larger sample sizes and more rigorous methodologies [50]. However, it remains uncertain what the correct sample size to overcome the complexity of the placebo effect.

The ethical implications of using placebos in clinical trials have been a topic of considerable debate. Regulatory agencies, including the World Medical Association and the FDA, have recommended avoiding placebo use in certain situations, primarily for ethical reasons. The Declaration of Helsinki, first adopted in 1964 and subsequently revised, emphasizes that placebos should not be used when effective treatments are available, as this could deny patients the best possible care [51]. The ethical argument against placebos hinges on beneficence and non-maleficence, ensuring that patients are not subjected to unnecessary harm or deprived of necessary treatment [48].

In trials where the placebo has a definitive perception or organic effect, the target drug will be considered less effective, a significant drawback of placebo–control studies. These studies raise an issue of ethics, as critically ill patients receiving a placebo will be disadvantaged [52].

The scientific community is not united on the value of placebo-controlled clinical trials, mainly because of the mindset among clinicians who want to see effectiveness in patients. An analogous situation arises when biosimilars already proven highly like the reference product are tested for comparative clinical efficacy. These studies cannot fail since any placebo effect is more than enough to differentiate the two products [53]. This trend demonstrates the need to educate clinicians who may not be aware of the placebo effect and prefer clinical trials against placebos, based on centuries of testing practices that have now been proven to need fresh concepts.

Testing new drugs against a placebo, where patients are given a blank pill, is similarly unethical and unnecessary as it reduces the effectiveness of the conclusion in various cases. There is also a dire need to educate clinicians about comparative clinical trials since drug developers most often conduct these trials to discuss these data with clinical practitioners primarily concerned with seeing proof of efficacy in patients.

### 6.2. Study Power

The FDA requires a study power of 80% and *p* < 0.05 for comparative clinical trials such as those conducted to prove biosimilarity [35,53]; however, in almost all such studies, the study power was significantly low, resulting in all such studies being positive. While several other agencies like the MHRA and EMA consider such trials uncertain and unnecessary, the FDA must take a similar action.

### 6.3. Bioavailability

The development of drugs that are intended to reach specific parts of the body, such as the brain, are approved by the FDA without requiring proof of bioavailability at the site of action, resulting in hundreds of failed trials involving thousands of patients [54]; such patient abuse can be avoided if the FDA would require that there is a definite proof that the drug can be effective based on its expected bioavailability.

## 7. Conclusions

The FDA has been proactive in updating its suggestions on designing and conducting clinical trials; however, there is still a need for a comprehensive summary guideline that should incorporate several guidelines that the FDA has issued recently. The concept of decentralizing trials can significantly impact the practicality and utility of these trials; the only trials that should matter are those conducted in a Real-World Environment, and now such trials can be conducted widely with the engagement of medical practitioners. One ancillary impact of these policies is that some funding will reach out to many institutions, such as academic hospitals, that can always benefit from the billions of dollars spent on these trials. I suggest that all patient treatment facilities consider establishing an FDA-approvable structure to meet the requirements for data collection, integrity, and reporting to the FDA. This will bring remarkable financial benefits besides enabling faster approval of drugs.

The FDA should continue its efforts to harmonize many remaining elements of clinical trials, including the use of placebos in the trials to bring the most expensive component of drug development; with reduced cost, we may see more new drugs reaching patients. Additionally, the biosimilars should be exempted from efficacy testing and other drugs as neurological therapies be allowed only if the drug has demonstrated a reasonable chance of being successful based on its bioavailability to the target organ.

## Figures and Tables

**Table 1:** Approximations of the cost of clinical trials.

Type of Clinical Trial	Where Used Mostly	Relative Cost
Phase 0 (Exploratory Trials)	Early exploratory drug development in small populations (usually healthy volunteers)	Low
Phase I (Safety Trials)	Early-stage testing in healthy volunteers or patients with targeted diseases	Moderate
Phase II (Efficacy and Safety Trials)	Larger patient groups, focusing on determining efficacy and optimizing dosage	High
Phase III (Confirmatory Trials)	Large-scale trials across multiple centers to confirm efficacy and monitor side effects	Very High
Phase IV (Post-marketing Trials)	After regulatory approval, focused on long-term safety and effectiveness in broader populations	Moderate to High
Real-World Evidence (RWE) Trials	Post-approval trials using real-world data from clinical settings, including health records, registries, or insurance claims	Moderate to High
Decentralized Trials	Trials conducted using remote technologies (e.g., telemedicine, wearables) to collect data from patients outside clinical sites	Moderate
Adaptive Trials	Trials that allow for modifications in design or procedures based on interim results (used in various phases, often in oncology)	High to Very High
Multi-Regional Trials	Trials conducted across multiple regions or countries, especially useful for global drug development	Very High
Randomized Controlled Trials Integrated into Routine Clinical Practice (Pragmatic RCTs)	RCTs embedded in routine care settings to assess the effectiveness of interventions in a real-world clinical practice context	High
Observational Trials	Non-interventional studies observing outcomes in routine clinical practice without manipulating the study environment	Low to Moderate
Randomized Controlled Trials (RCTs)	Gold standard for testing the efficacy of interventions in controlled settings	Very High
Crossover Trials	Used in chronic conditions where patients switch between treatments during the trial	High
Single-Arm Trials	Often used for rare diseases where randomization is difficult	Moderate to High
Basket Trials	Common in oncology, used to test a drug across multiple cancer types with the same genetic mutation	High
Umbrella Trials	Typically used in oncology, testing multiple drugs for the same disease population	Very High
